# Is Osteoarthritis a State of Joint Dysbiosis?

**DOI:** 10.3390/antibiotics14060609

**Published:** 2025-06-15

**Authors:** Mincong He, Frank Kolhoff, Michael A. Mont, Javad Parvizi

**Affiliations:** 1 The Third Affiliated Hospital, Guangzhou University of Chinese Medicine, Guangzhou 510000, China; min-cong.he@hotmail.com; 2 International Joint Center, Acibadem Maslak Hospital, Istanbul 34303, Turkey; frank_k@live.nl; 3 Department of Orthopedics, Spaarne Gasthuis, 2134 TM Hoofddorp, The Netherlands; 4 Rubin Institute for Advanced Orthopaedics, Baltimore, MD 21215, USA; rhondamont@aol.com

**Keywords:** osteoarthritis, joint microbiome, gut microbiota, gut–joint axis, contaminants

## Abstract

Osteoarthritis (OA) has traditionally been defined as a degenerative joint disease driven by mechanical wear, aging, and metabolic disturbances. However, emerging evidence suggests that joint dysbiosis, a dysregulation in the joint microbiome, may play an important role in OA pathogenesis. This review explores the mechanisms linking dysbiosis to OA. We examine the presence and origin of joint dysbiosis, also highlighting the gut–joint and oral–joint axes as potential routes for microbial translocation. However, challenges remain in distinguishing causation from correlation and addressing microbial contaminants in microbiome studies. Future research should prioritize longitudinal studies and multiomics integration to elucidate the complex interplay between microbial communities and joint health.

## 1. Introduction

Osteoarthritis is one of the leading causes of disability worldwide, particularly affecting approximately 37% of individuals aged 60 years and older [1]. Although OA is considered an age-related disease, studies have shown that younger patients can also be afflicted [2,3]. Moreover, OA poses a major socioeconomic burden globally, with reports estimating that more than 500 million people suffer from OA and treatment costs reaching up to 2.5% of GDP [4,5]. As the population ages, the prevalence is expected to continue rising [6]. Risk factors, including older age, women, being overweight or obese, having a knee injury, and occupational factors, have been associated with the occurrence of disease [1]. However, despite the insights provided by traditional etiological models, there are notable gaps in our understanding of OA. The heterogeneity in presentation and progression remains a challenge. Not all individuals who have mechanical risk factors, such as joint injuries or repetitive stress, develop OA, and the rate of disease progression varies markedly among patients [7]. Furthermore, some cases lack clear mechanical or metabolic triggers, suggesting the involvement of alternative pathways. There is ample evidence to implicate the involvement of other factors, including environmental influences, joint inflammation factors, and the microbiome, in OA etiology. The limitations of current models highlight the need for new paradigms to explain the variability and individual differences. The emerging hypothesis of joint dysbiosis, a dysregulation in the joint microbiome, offers a potential avenue for addressing these gaps in understanding.

## 2. Current Perspective on Etiology and Pathophysiology of OA

OA is a degenerative joint disease that has historically been attributed to a combination of mechanical and biochemical factors [8,9]. The traditional view characterizes OA as the cumulative effects of mechanical stress, age-related changes, and chronic inflammatory conditions that lead to the breakdown of articular cartilage and subsequent joint damage [10]. Primarily, the wear and tear of the joint was seen as the leading cause of the degeneration process associated with OA. However, age-related biochemical alterations were found, revealing concomitant changes to cartilage homeostasis [10]. In OA conditions, autolytic enzymes, including matrix metalloproteases (MMPs) and aggrecanases, are upregulated, affecting the extracellular matrix, resulting in cartilage loss. Also, inflammatory cytokines, including interleukin-1 and tumor necrosis factor α (TNF α), are elevated in OA joints [11,12]. Their presence induces more enzyme release by chondrocytes and synovial cells, resulting in promoted inflammation, which acts as a vicious circle. Transforming growth factor beta (TGF β) is a cytokine that plays a complex role in the process of OA. The TGF β supports cartilage matrix and chondrocytes and protects against these autolytic enzymes and proteinase pathways [13]. Yet, age-related adaptations in TGF β signaling are found in OA patients, affecting chondrocyte formation and resulting in degenerated cartilage matrix and osteophyte formation [14]. This emphasizes that cumulative (age)-altered biochemical and inflammatory disorders are contributory to OA.

## 3. Emerging Hypothesis: Dysbiosis as a Driver of OA

The concept of dysbiosis, or microbial imbalance, is increasingly being considered as a potential contributor to joint inflammation and degeneration in OA [15,16]. This hypothesis suggests that alterations in the microbial communities within the body, including the gut and potentially the joint itself, can influence the initiation and progression of OA. Parallels can be drawn with other inflammatory conditions, such as rheumatoid arthritis (RA) and spondyloarthritis, where dysbiosis is a well-established factor [17,18,19]. The microbiota, particularly gut microbiota, can influence OA through various mechanisms. The gut–joint axis describes how gut-microbiota-related metabolites, such as short-chain fatty acids (SCFAs) and lipopolysaccharides (LPS), can affect joint health [7,20]. Alternatively, microbial products, such as LPS, can activate Toll-like receptors (TLRs) and nuclear factor kappa-B (NF-κB) signaling pathway, promoting synovitis and cartilage breakdown [21]. In the oral joint axis, *Porphyromonas gingivalis*, a bacterium associated with periodontal disease, has been shown to exacerbate arthritis in animal models, potentially through the generation of citrullinated proteins [22]. These research findings suggest that dysbiosis may play an important role in the pathogenesis of OA, warranting further investigation and exploration of microbiota-targeted therapies.

## 4. Evidence for the Existence of a Joint Microbiome

The concept of a “joint microbiome” refers to the community of microorganisms, including bacteria, fungi, and viruses, that reside within the joint space [23,24]. This challenges the traditional view of joints as sterile environments and suggests that these microbial communities may play a role in joint health and disease, particularly in the context of OA. Recent studies employing different DNA/RNA sequencing techniques, such as 16S rRNA gene sequencing or Next-Generation Sequencing (NGS), have identified microbiomes and, in some cases, live organisms within the synovial fluid (SF) and joint tissues (Table 1) [24,25,26,27,28,29,30,31,32,33,34]. In 2009, Siala et al. utilized a broad-range bacterial polymerase chain reaction (PCR), amplifying a 1400 bp fragment from the 16S rRNA gene and identifying bacterial DNA in 20 of the 27 SF samples (74.1%) [30]. Mixtures of bacterial nucleic acids, including *Stenotrophomonas maltophilia* and Shigella species were mainly detectable in the SF samples from patients who have OA. In another study, Témoin et al. detected bacterial DNA in the SF samples of five patients out of a total of thirty-six patients (13.9%): two who had RA (one native and one failed prosthetic joint) and three who had OA (one native and two failed prosthetic joints). Of these five patients, two were diagnosed with periodontitis and had identical bacterial clones (*Fusobacterium nucleatum* and *Serratia proteamaculans*) detected in both the SF and the dental plaque samples. *Fusobacterium nucleatum* was the most prevalent, detected in four of the five positive samples [32]. Tarabichi et al. detected six positive samples in a total of 17 samples (35.3%) with NGS (including 16S rRNA and Internal Transcribed Spacer (ITS) for both bacteria and fungi) in the OA group, whereas all positive samples originated from tissue, and swabs and fluid were all culture-negative [31]. In three cases, the predominant organism originated from the Proteobacteria phylum, representing 98, 66, and 50% of the samples. In other samples, organisms from the Fusobacteria and Actinobacteria phyla were detected with high percentages [31]. Zhao et al. used 16S rRNA gene amplicon sequencing to assess bacterial nucleic acid communities in synovial tissue and SF samples from patients who have RA and OA. They found that Atopobium, Phascolarctobacterium, and *Rhodotorula mucilaginosa* were more abundant in synovial tissues of OA compared to RA. *Bacteroides caccae* was more abundant in the SF of OA. The analysis of a large number of sequences revealed the presence of bacterial DNA from more than one bacterial species in the SF of all patients studied [34]. The results from Zhao et al. were markedly different from research findings from other centers in the United States (such as the studies by Tsai et al., Borsinger et al., and Fernandez et al. [23,24,28]). These differences may be due to geographic or dietary effects [35,36]. In the study of Torchia et al., *Escherichia coli* was found to be the most common bacterium in naïve knees [33]. This finding implicated the idea of a link between the gut microbiome and knee pathology [37,38]. The presence of fungal species like *Cladosporium herbarum* and *Alternaria alternata* in the joint has also been reported [33]. In the study by Dunn et al., Clostridium and phylum Bacteroidetes were abundant in the non-OA control group, while Betaproteobacteria and order Burkholderiales were seen in the OA group using 16S rRNA sequencing. Moreover, this finding was also verified by an in vivo study using a mouse OA model [26]. The latter study suggested that the degeneration of cartilage increases its exposure directly to the blood and to the inflammatory mediators in the systemic circulation, which makes it more vulnerable to the faster and higher accumulation of bacterial DNA. Tsai et al. aligned RNA-sequencing data from OA patient synovial tissue to a library of microbial reference genomes to identify microbial reads indicative of microbial abundance. They found that microbes correlated with OA are related to the dysregulation of two main functional pathways: increased inflammation-induced extracellular matrix remodeling and decreased cell signaling pathways crucial for joint and immune function [24]. In the study by Fernandez et al., not only were healthy non-OA control cases included, but the SF from the non-OA contralateral knee joints of the patients was also analyzed using 16S rRNA sequencing [28]. Interestingly, Cutibacterium, Staphylococcus, and Paracoccus species constituted over 75% of the bacterial species in the healthy control group, while the abundance of Proteobacteria, which is a major member of the gut microbiome in the neonatal period during the gut microbiome instauration, was significantly higher in the OA group [39,40]. Since the expansion of the Proteobacteria phylum can affect nonintentional tissue, this finding may reveal the existence of a cause-and-effect relationship between Proteobacteria abundance and OA in the knee joint. In the multicenter study by Goswami et al., the five most prevalent genera in the arthritic joints were Escherichia, Cutibacterium, Staphylococcus, Acinetobacter, and Pseudomonas [29]. The study by Elsawy et al., instead of using RNA sequencing, used real-time PCR to detect specific bacterial phyla in the SF sample from knee OA [27]. Using ultrasound screening, decreased lateral femoral cartilage thickness was associated with the increased abundance of Firmicutes.

We have to note that these 11 studies have reported positive rates ranging from as low as 3.8% to as high as 100% [25,27]. The wide variation in reported positive rates across these studies may be attributable to a combination of factors, including differences in sample collection, DNA extraction, amplification methodologies, contamination issues, data analysis pipelines, and patient-related characteristics. Variability in detection rates may arise at the very first step: aspiration prior to the arthrotomy versus aspiration prior to the skin incision. Theoretically, collecting SF samples before arthrotomy but after the skin incision can reduce the contamination from skin flora. Thus, it may be one of the reasons that higher positive rates are reported in studies from Siala et al. (74.1%) and Elsawy et al. (100%) [27,30]. Storage conditions and processing protocols can also influence the level of exogenous contamination. Studies employing rigorous DNA extraction techniques and immediate processing tend to have lower contamination levels, whereas less controlled environments may lead to false-positive signals or artificially inflated detection rates [41]. Moreover, the employment of PCR-based methods introduces amplification biases that are exacerbated under conditions of low template concentration. Universal primers used for 16S rRNA amplification may differ in their specificity and coverage of bacterial taxa. Additionally, excessive PCR cycling can inadvertently amplify contaminants, leading to false-positive detections. In contrast, protocols with fewer cycles or the use of high-fidelity polymerases may yield lower detection rates but provide a more accurate representation of the genuine microbial population [42]. The depth of sequencing and the bioinformatic pipelines in NGS used for quality control and contaminant filtering also play critical roles in determining whether low-abundance sequences are designated as true positives or dismissed as background noise [43]. Also, aside from technical issues, inherent differences in study populations and clinical conditions can influence microbial detection rates. Patient-related factors such as personal and dietary habits, geographical distribution, and ethnic differences may affect the microbial load and diversity [44,45]. Moreover, the definition of a “positive” result—often based on arbitrary thresholds of read counts or relative abundance—varies across studies, contributing further to the observed heterogeneity.

Generally, the aforementioned studies provide evidence for the existence of a joint microbiome, although the composition and functional roles of these microbial communities are still under investigation. The composition of the joint microbiome appears to differ between healthy joints and those affected by OA. These differences suggest that dysbiosis, or an imbalance in the joint microbiome, may contribute to the pathogenesis of OA or that the development of OA may alter the microbiome composition of a joint.

## 5. Contaminants in Joint Microbiome

Distinguishing between the resident microbiota of the joint and external contaminants is crucial for accurately understanding the role of dysbiosis in OA. Contaminants can be introduced during surgical interventions, such as joint aspiration or arthroplasty, or through environmental factors [33]. If not properly identified and accounted for, these contaminants can confound the results of microbiome studies and lead to inaccurate conclusions about the composition and function of the joint microbiome [46].

Synovial fluid, synovial tissue, and cartilage tissue are low-biomass samples compared to fecal samples. Low-biomass samples present unique challenges in microbial high-throughput sequencing studies. Synovial fluid and cartilage tissue are traditionally considered to reside in relatively sterile or low-microbial-load environments. In such samples, the endogenous microbial DNA is often near the detection limit of sequencing platforms. Any detected microbial DNA might either derive from transient exposure or reflect laboratory-based contamination rather than a resident microbial community. Thus, contaminant DNA can represent a major proportion of the total sequence read count. This low signal-to-noise ratio substantially indicates the determination of true microbial constituents versus false positives [41]. It is not only laboratory-based contamination, PCR amplification bias, sequencing cross-contamination, and data analysis and bioinformatic processing that can cause a false positive in low-biomass samples [47,48].

Researchers employ several strategies to avoid contamination and differentiate between resident microbiota and external contaminants (Table 2). One approach is to use stringent sterile techniques during sample collection and processing to minimize the introduction of external microorganisms [46]. Another strategy is to include negative controls, such as sterile water or media, in the experimental workflow to identify potential contaminants. If the same microorganisms are found in the negative controls and the joint samples, it is likely that they are contaminants rather than true residents of the joint [33]. Another method involves analyzing the microbial composition of different joint tissues and fluids to identify core microbial communities that are consistently present across individuals. These core communities are more likely to represent the resident microbiota of the joint, while microorganisms that are only sporadically detected are more likely to be contaminants. For example, some studies have focused on identifying microbial DNA signatures in cartilage samples and comparing them to those found in SF or other joint tissues [26]. Furthermore, comparing the microbial profiles of patients undergoing joint surgery with those who have not undergone surgery can help identify microorganisms that are specifically associated with surgical interventions. Luo et al. found that the preoperative presence of microorganisms in knee joints of RA patients is common (24.5%), and intraoperative synovial tissue culture is valuable for the diagnosis of this condition and in the selection of antibacterial treatment [49]. By carefully considering the potential sources of contamination and employing appropriate control measures, researchers can gain a more accurate understanding of the composition and function of the resident joint microbiome and its role in OA pathogenesis.

## 6. Hypotheses on Microbial Origins

### 6.1. Gut–Joint Axis

The gut–joint axis refers to the bidirectional communication between the gut microbiome and the joints [20,53]. This axis suggests that alterations in the gut microbiome, or gut dysbiosis, can indirectly, or even directly, influence joint health and contribute to the development of OA. The gut microbiome produces a variety of metabolites, SCFAs, LPS, and peptidoglycans, which can enter the bloodstream and circulate throughout the body [54]. Some of these bacterial metabolites, such as LPS, can promote inflammation and immune activation. When these metabolites reach the joint space, they can activate immune cells, such as macrophages and synovial fibroblasts, leading to the production of pro-inflammatory cytokines and matrix-degrading enzymes [55,56]. This chronic inflammation can contribute to cartilage degradation and the development of OA.

Another proposed mechanism involves the translocation of gut pathogens to the joint space [57]. In individuals who have gut dysbiosis, the intestinal barrier may become compromised, allowing organisms or their byproducts to leak from the gut into the bloodstream. Microbiota from the circulation enters the joint through the subchondral vascular channels of the calcified zone of the cartilage and the zones of neoangiogenesis, which are formed at the osteochondral junction of the OA cartilage, creating a state of “focal dysbiosis” [58]. These pathogens can then travel to the joints and trigger an immune response [57].

### 6.2. Oral–Joint Axis

The oral–joint axis proposes a link between oral health, particularly periodontal disease, and joint health [32]. Periodontal disease is a chronic inflammatory condition caused by the bacterial infection of the gums and supporting tissues of the teeth. One potential mechanism involves the translocation of periodontal pathogens from the oral cavity to the joint space. Microorganisms from the oral cavity can enter the bloodstream during daily activities such as chewing and brushing, as well as during dental procedures. These bacteria can then travel to the joints and trigger an immune response. Témoin et al. found bacterial DNA in the SF samples from patients who have arthritis and failed prosthetic joints and identified identical bacterial clones (*Fusobacterium nucleatum* and *Serratia proteamaculans*) in both the SF and the dental plaque samples of some patients [32]. This finding suggests the possibility of organisms translocating from the periodontal tissue to the synovium.

Another potential mechanism involves the systemic inflammatory effects of periodontal disease. Chen et al. assessed the oral microbiome in saliva samples from patients who have RA, patients who have OA, and healthy subjects using 16S rRNA gene amplicon sequencing and identified eight oral bacterial biomarkers to differentiate RA from OA [59]. The oral–joint axis is supported by epidemiological studies that have found an association between periodontal disease and OA [32]. These studies suggest that individuals who have periodontal disease may be at a higher risk of developing OA, although further research is needed to confirm this association and elucidate the underlying mechanisms.

### 6.3. Post-Surgical and Environmental Contaminants

Surgical procedures, such as joint arthroplasty or arthroscopy, can potentially deliver microorganisms into the joint space, potentially leading to post-surgical infections or contributing to joint dysbiosis [60]. Environmental factors, such as exposure to certain microorganisms or toxins, may also play a role in the development of joint dysbiosis. During surgery or invasive procedures, microorganisms from the skin, surgical instruments, or the operating room environment can enter the joint space. While sterile techniques are employed to minimize contamination, it is not always possible to prevent the introduction of microorganisms entirely. Environmental factors may also contribute to joint dysbiosis. Exposure to certain microorganisms in the environment, such as bacteria or fungi, may lead to their active colonization of the joint space [46].

Further research is needed to fully understand the role of post-surgical and environmental contaminants in the development of joint dysbiosis and OA. Identifying specific environmental or procedural conditions that are more likely to lead to dysbiosis could help develop strategies to prevent or mitigate these risks.

## 7. Potential Mechanisms Linking Dysbiosis and Pathophysiology of OA

Understanding the mechanisms by which joint dysbiosis contributes to the pathophysiology of OA is crucial. However, to our knowledge, there are no studies that reveal a direct interactive mechanism between intra-articular bacteria and local tissues to date. Dysbiosis-induced immune disorder may potentially be the key factor that can disrupt joint homeostasis and accelerate OA progression.

**Figure 1:**
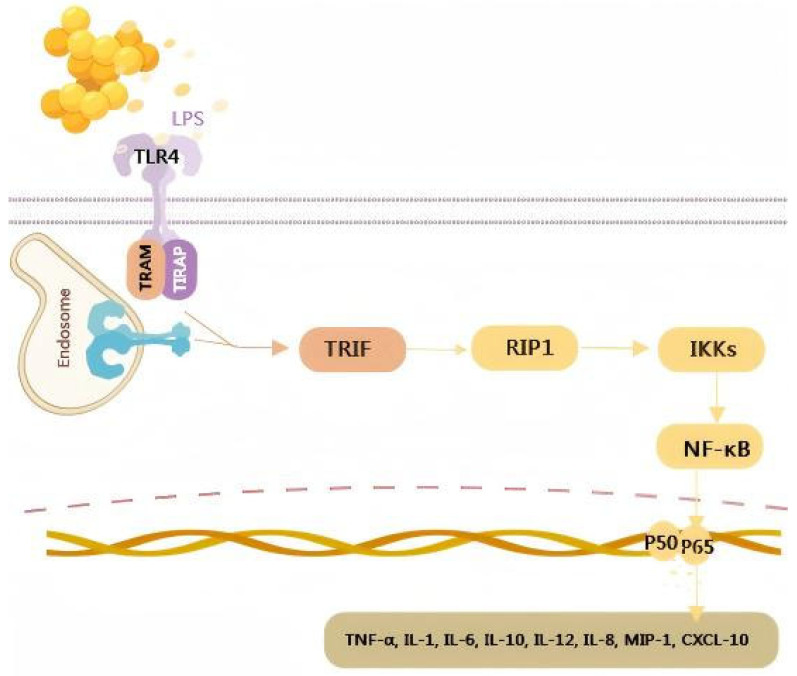
LPS/TLR4/NF-κB pathway in the synovium.

Microbiota-induced inflammatory pathways refer to the activation of immune responses within the joint environment triggered by alterations in the microbiome. These pathways play a major role in OA pathology by promoting inflammation and cartilage degradation. LPS, a component of the outer membrane of Gram-negative bacteria, can initiate inflammatory responses through the TLR4/NF-κB pathway in the synovium [61]. The interaction between LPS and TLR4 triggers a series of intracellular signaling events, leading to the activation of NF-κB, a transcription factor that regulates the expression of pro-inflammatory cytokines (Figure 1). Specific immune cells, such as macrophages and T-cells, participate in the chronic inflammation characteristic of OA [62]. This inflammatory cascade may affect the synovial membrane and joint tissues, leading to structural changes and pain. Chronic inflammation of the synovial membrane can result in synovial hyperplasia, increased vascularity, and infiltration of immune cells. These changes can further contribute to the production of inflammatory mediators and MMPs, which degrade the cartilage matrix and promote bone remodeling, ultimately leading to the structural damage observed in OA.

## 8. The Effect of Antibiotic-Induced Gut Microbiome Dysbiosis on OA

There is some controversy regarding the use of antibiotics in the treatment of arthritis. According to previous studies, antibiotic treatment can destroy the commensal balance of the human microbiome, which affects intestinal integrity and promotes the leakage of bacterial metabolites such as LPS into the circulation [63]. An increased level of serum LPS is associated with knee osteophyte severity and joint pain [7]. However, recent studies have investigated the effect of antibiotic administration in OA. Mendez et al. demonstrated that antibiotic treatment (1.0 g/L ampicillin and 0.5 g/L neomycin in drinking water), which induced gut microbiome dysbiosis, was associated with reduced articular cartilage damage in mice subjected to dynamic tibial compressive overload following anterior cruciate ligament injury. A likely explanation for this result is that the gut microbiome dysbiosis led to reduced LPS serum levels and alleviated the inflammatory response, including TNF α and Interleukin-6 [64,65]. In a canine model of osteoarthritis induced by anterior cruciate ligament transection, the oral administration of doxycycline (50 mg, twice daily) significantly alleviated the progression of arthritis [66]. Furthermore, these results were further validated in a randomized, double-blind, and placebo-controlled clinical trial with the intervention measures of 100 mg doxycycline or placebo twice a day for 30 months [67]. However, this evidence may not yet be sufficient to demonstrate that antibiotic treatment has a mitigating effect on the progression of osteoarthritis. While these findings suggest the attributional effects of antibiotics, the long-term impact of antibiotic administration on the host’s healthy microbiome should not be neglected. Antibiotic use can alter both the diversity and functional capacity of the microbiome, potentially compromising host health.

## 9. Current Controversies and the Future

In the context of OA and other microbiota-related diseases, it is crucial to differentiate between causation and correlation. Correlation indicates a statistical association between dysbiosis and OA, meaning that changes in the microbiome are observed alongside the disease. Causation, on the other hand, implies that dysbiosis directly contributes to the development or progression of OA. Evidence exists to support dysbiosis as a potential cause of OA. For example, *Porphyromonas gingivalis* oral infection in mice leads to gut dysbiosis and aggravates arthritis, subsequently increasing the production of citrullinated proteins and inflammatory cytokines [22]. Similarly, fecal microbiota transplantation from metabolically compromised human donors into germ-free mice accelerates OA development after joint injury, suggesting a direct causal link between gut microbiota composition and OA severity [57]. However, counterarguments suggest that the observed changes in the joint microbiome may simply reflect the systemic effects of advanced OA, such as the inflammatory processes and tissue barrier damage, rather than being its cause. It is possible that the altered joint environment in OA, characterized by inflammation and tissue damage, creates an opportunity for opportunistic colonization by certain microbes [68]. The presence of bacterial DNA in SF and joint tissues does not necessarily indicate a causal role in OA; it could be a consequence of the disease process itself [30,34]. Distinguishing between pathogenic species and commensal organisms within the joint microenvironment is essential. While some microbes may actively promote inflammation and cartilage degradation, others may be harmless residents or even exert protective effects. Microorganisms such as streptococcus species have been associated with increased knee pain and local inflammation, while other commensal bacteria might play a role in maintaining joint homeostasis [56]. Future research may need to establish possible relationships between bacteria and OA on both metabolic and clinical outcomes, including patient-reported outcome measures, radiographic findings, or MRI results.

Microbiome analyses face several challenges, with the introduction of contaminants perhaps being the most important concern. Common sources of contamination include laboratory reagents, sampling procedures, and environmental factors. These contaminants can introduce extraneous microbial DNA, leading to inaccurate results and misinterpretations of the true microbial composition of the sample. Detecting low-biomass microbial communities in SF or cartilage poses another unique difficulty. The limited amount of microbial DNA present in these samples makes them particularly vulnerable to analytical errors. Furthermore, the presence of host DNA and other biological molecules can interfere with DNA extraction and sequencing, further complicating the analyses.

Identifying microbial biomarkers for prognostication may be a direction that microbiome research may need to pursue in the future, as current diagnostics fail to identify early OA progression. Preliminary findings link specific microbial signatures to different stages or severities of OA. For example, certain bacterial species may be more abundant in patients who have early-stage OA, while other organisms may be associated with more advanced disease. Similarly, specific bacterial metabolites may be indicative of cartilage degradation or inflammation [56]. These findings could provide novel strategies for the prevention of OA progression.

Thorough perspective studies into the revelation interplay among joint dysbiosis, inflammation, and OA severity through longitudinal and interventional studies, perhaps with dietary control, are crucial for exploring potential precision microbial therapies and fully understanding the complex relationship between microbial communities and OA pathogenesis. To translate these initial insights into targeted interventions, prospective longitudinal and interventional studies into the relational interplay among joint dysbiosis, inflammation, and OA severity are crucial for exploring potential precision microbial therapies and fully understanding the complex relationship between microbial communities and OA pathogenesis.

## 10. Conclusions

Traditional perspectives have dominated both the research and clinical approaches to OA, emphasizing the role of joint biomechanics, cartilage integrity, and systemic metabolic factors in disease progression. However, emerging evidence suggests that OA may also be markedly influenced by joint dysbiosis, challenging and expanding the conventional etiological models of the disease. Key findings from various studies have demonstrated the presence of diverse microbial populations in the SF and joint tissues of OA patients, indicating that these microorganisms might play an active role in disease pathogenesis. These new findings indicate that beyond mechanical and metabolic factors, the microbial environment within the joint plays a crucial role in modulating inflammatory pathways and contributing to cartilage degradation. Such a renewed concept opens up novel theory for investigating the underlying mechanisms of OA, emphasizing the need to explore microbial interactions and their impact on joint health. Mechanical wear and metabolic disturbances are challenging to address, while microbial populations can be influenced through dietary interventions, probiotics, antibiotics, and other microbiome-targeted therapies. This distinction emphasizes the potential for more personalized and adaptable treatment, such as probiotic and prebiotic therapies or fecal microbiota transplantation (FMT), that accounts for the unique microbial signatures of individual patients. However, despite the promise of these microbiome-based interventions, several challenges must be addressed. Factors like genetics, diet, and the existing microbial communities of the individual can influence treatment outcomes [7]. Personalized approaches that tailor microbiome therapies to the unique microbial profiles of patients may help overcome this variability and enhance the effectiveness of treatments.

Additionally, clinical trials are essential to establish evidence-based guidelines for the widespread clinical use of microbiome-based therapies in the treatment of OA. These trials may focus on identifying optimal intervention strategies, dosages, and treatment durations, as well as assessing potential side effects and long-term benefits.

Furthermore, there is a growing demand for developing frontier techniques to support microbiome research and its clinical applications. The standardization of sample collection, the rigorous use of negative controls, optimized DNA extraction methods, and refined bioinformatic analysis are essential. Future investigations should emphasize the integration of robust quality control measures to distinguish between true microbial signals and technical artifacts. This includes investing in advanced sequencing technologies with low false positive rates in low-biomass samples, bioinformatics tools, and interdisciplinary training programs that consist of healthcare professionals who have the knowledge and skills necessary to utilize microbiome-based diagnostics and therapeutics effectively.

In conclusion, the integration of joint dysbiosis and microbiome-based innovations offers a promising field for OA research. Overcoming the challenges associated with individual variability and the need for clinical evidence will be crucial in realizing the widespread application of these innovations. As research continues to illuminate the intricate connections between the microbiome and joint health, the adoption of microbiome-based approaches in clinical settings is poised to transform the landscape of OA treatment, offering patients more effective and personalized care options.

## Figures and Tables

**Table 1:** Clinical evidence of joint microbiome.

Author (Year)	Joint	Sex of Patients (M/W)	Case Numbers	Sample	Age	Detection Method	Positive Rate	Finding (in OA Subgroup)
Siala et al. (2009) [30]	Knee	16/11	*n* = 27 (RA = 7 ReA = 5 OA = 6 UA = 9)	SF	58 (44 to 70)	16S rRNA	20/27 74.1%	The first study using the full-length 16S rRNA gene to detect bacterial DNA in SF. Mixtures of bacterial nucleic acids, including *Stenotrophomonas maltophilia*, and Shigella species mainly, were detectable in the SF samples from patients who have OA.
Temoin et al. (2012) [32]	Knee	9/27	*n* = 36 (RA = 11 OA = 25)	SF	61.6 (45 to 84)	16S rRNA	5/36, 13.9%	*Fusobacterium nucleatum* was the most prevalent in positive samples in the OA group. The findings of bacterial DNA in the SF suggest the possibility of organisms translocating from the periodontal tissue to the synovium.
Tarabichi et al. (2018) [31]	Knee and Hip	-	*n* = 82; Revision arthroplasties (*n* = 65) primary arthroplasties (*n* = 17)	SF, ST, and swabs	/	NGS (16S rRNA and ITS)	6 of 17 (in the primary arthroplasty group)	Proteobacteria phylum was found to represent 98, 66, and 50% of the three bacteria. Fusobacteria and Actinobacteria phyla were detected with high percentages in other samples.
Zhao et al. (2018) [34]	Knee	31/152	*n* = 183 (RA = 125 OA = 58)	SF and ST	Not mentioned	16S rRNA	Not mentioned	The most abundant phyla in OA synovial samples were Proteobacteria (ST, 55.1%; SF, 39.1%), Bacteroidetes (ST, 20.4%; SF, 29.4%), and Firmicutes (ST, 17.0%; SF, 24.0%).
Torchia et al. (2019) [33]	Knee	19/21	*n* = 40	SF	65.8 ± 9.38	NGS (16S rRNA and ITS)	12/40, 30.0%	The most common organism identified in the native knee was *Escherichia coli,* implicating the idea of a link between the gut microbiome and knee pathology. Also, fungi (*Cladosporium herbarum and Alternaria alternata*) were found by NGS.
Dunn et al. (2020) [26]	Knee and Hip	15/16	*n* = 31 (OA = 21 Con (cadaver) *n* = 10)	Cartilage	OA: 59 ± 2, Control: 68 ± 4	16S rRNA	-	Clostridium and phylum Bacteroidetes were found to be significantly increased in the control group, and Betaproteobacteria and order Burkholderiales were increased in the OA group. This finding was also verified by *in vivo* research in a mouse OA model.
Tsai et al. (2020) [24]	Knee	11/13	*n* = 24; OA = 14; Con = 10	SF	OA = 50.2 (19 to 69), Control = 37.8 (22 to 63)	Separated microbe-specific reads from RNA-seq GSE89408;	Not mentioned	Pseudomonas species was found to be the most abundant species in the OA patients among 43 differential microbes compared to the control group. Parts of them (9/43) were revealed to relate to increased inflammation-induced extracellular matrix remodeling and decreased cellular communication essential for joint function and immune function in the OA pathological process.
Borsinger et al. (2023) [25]	Knee	18/22	*n* = 80 (TKA knee = 50 Con = 30	SF, ST, and swabs (combined)	67 (41 to 84)	NGS (16S rRNA and ITS)	3/80, 3.8%	*Cutibacterium acnes* was the most common in the OA group.
Fernandez et al. (2023) [28]	Knee		*n* = 65 (OA = 14 Con = 15 NOA Contra = 10 Aseptic revision = 14 PJI = 12	SF	61 (50 to 66)	16S rRNA	55.8%	Staphylococcus and Paracoccus species consisted of 75% of bacterial abundance in the healthy control group, while the abundance of Proteobacteria, which is a major member of the gut microbiome in the neonatal period during the gut microbiome instauration, was significantly higher in the OA group. It may reveal the existence of a cause–and-effect relationship between Proteobacteria abundance and OA in the knee joint.
Goswami et al. (2023) [29]	Hip or knee	-	*n* = 117 (from 13 academic institutions)	SF, ST, and swabs	Not mentioned	16S rRNA	113/117, SF = 38%; Swabs = 43% ST = 36%	Representing the largest characterization of the composition of native joint microbiota to date. The first five most prevalent genera were Escherichia, Cutibacterium, Staphylococcus, Acinetobacter, and Pseudomonas.
Elsawy et al. (2024) [27]	Knee	11/29	*n* = 40	SF	60 (43 to 72)	RT-PCR (for specific bacterial phyla)	40/40, 100%	The most abundant bacterial phyla were Firmicutes (63.6%), Actinobacteria (24.1%), and Proteobacteria (11.5%). Firmicutes was associated with decreased lateral femoral cartilage thickness.

Abbreviations: ReA: Reactive Arthritis, UA: Undifferentiated Arthritis, SF: synovial fluid, ST: synovial tissue, ITS: Internal Transcribed Spacer, NGS: Next-Generation Sequencing, Con: control, NOA Contra: non-OA contralateral knee joints, RT-PCR: real-time polymerase chain reaction.

**Table 2:** Contaminant control strategies in different studies.

Author (Year)	Detection Method	Positive Rate	Contaminant Control Strategies
Siala et al. (2009) [30]	16S rRNA	20/27 74.1%	The ST samples were collected from the knee joint using the biopsy procedure without skin incision after skin surface disinfection. Additional precautionary measures were taken to prevent DNA contamination during DNA extraction and manipulation [50].
Temoin et al. (2012) [32]	16S rRNA	5/36, 13.9%	Sterile saline solution was aspirated out of a surgical basin and transferred to sterile microcentrifuge tubes in the same manner as control samples after synovial fluid aspiration as control samples.
Tarabichi et al. (2018) [31]	NGS (16S rRNA and ITS)	Six of 17 (in the primary arthroplasty group)	The ST samples were collected from the knee using an 18-gauge needle prior to arthrotomy. Deep tissue specimens were taken from the synovium and medullary canals. Swabs from the medullary canal of the femur and tibia were obtained from knees.
Zhao et al. (2018) [34]	16S rRNA	Not mentioned	The SF samples were collected aseptically during therapeutic aspiration from knee joints. Sample collection, reaction mixture controls, and an environmental control (a tube filled with sterile phosphate-buffered saline was left open for the duration of the surgical procedure and then processed in parallel with the samples) were included for negative control.
Torchia et al. (2019) [33]	NGS (16S rRNA and ITS)	12/40, 30.0%	The SF was aspirated from the knee joint prior to the arthrotomy, but after the skin was incised. Four sterile water controls were also sent as part of the protocol. Four sterile water samples from a container on the operative field at the time of synovial fluid and tissue sampling were collected as negative controls.
Dunn et al. (2020) [26]	16S rRNA	-	Identifying microbial DNA signatures in cartilage samples and comparing them to those found in SF or other joint tissues.
Tsai et al. (2020) [24]	Separated microbe-specific reads from RNA-seq GSE89408;	Not mentioned	Employing Spearman’s correlation to compare individual microbe abundance levels to total microbial reads based on the idea that contaminant microbes would reveal a similar microbial abundance in all samples, regardless of the size of the tissue. The microbe was considered to be of contamination if the regression line was vertical (no contaminant microbes were found).
Borsinger et al. (2023) [25]	NGS (16S rRNA and ITS)	3/80, 3.8%	The SF samples were aspirated from the knee joint prior to the arthrotomy but after the skin was incised. Three negative controls were run for every 92 samples in the DNA extraction procedure.
Fernandez et al. (2023) [28]	16S rRNA	55.8%	SF samples were aspirated from the knee joint prior to the arthrotomy but after the skin was incised. The bacteria that are well-known contaminants of chemical reagents (such as *Bradyrhizobium* species) were discarded from the sequencing results [51,52].
Goswami et al. (2023) [29]	16S rRNA	113/117, SF = 38%; Swabs = 43% ST = 36%	The SF samples were obtained using an 18-gauge needle before arthrotomy. Samples were compared to negative controls with no PCR template.
Elsawy et al. (2024) [27]	RT-PCR (for specific bacterial phyla)	40/40, 100%	Sterile distilled water as RT-PCR control samples.

Abbreviations: SF: synovial fluid, ST: synovial tissue, ITS: Internal Transcribed Spacer, NGS: Next-Generation Sequencing, RT-PCR: real-time polymerase chain reaction.

## Abbreviations

The following abbreviations are used in this manuscript:

OA	Osteoarthritis
MMPs	Metalloproteases
TNF α	Tumor necrosis factor α
TGF β	Transforming growth factor beta
RA	Rheumatoid arthritis
SCFAs	Short-chain fatty acids
LPS	Lipopolysaccharides
TLRs	Toll-like receptors
NF-κB	Nuclear Factor kappa-B
NGS	Next-generation sequencing
SF	Synovial fluid
PCR	Polymerase chain reaction
ITS	Internal Transcribed Spacer
